# The link between psychological distress and survival in solid tumor patients: A systematic review

**DOI:** 10.1002/cam4.5200

**Published:** 2023-01-05

**Authors:** Kayla N. Roche, Diane Cooper, Terri S. Armstrong, Amanda L. King

**Affiliations:** ^1^ Neuro‐Oncology Branch National Cancer Institute, National Institutes of Health Bethesda Maryland USA; ^2^ National Institutes of Health Library Bethesda Maryland USA

**Keywords:** cancer, distress, patient‐reported outcomes, survival, tumors

## Abstract

**Purpose:**

Research has demonstrated that solid tumor patients experience high levels of psychological distress at the time of diagnosis. While distress has been associated with many adverse clinical outcomes, little is known about how this symptom may influence the disease trajectory for cancer patients, affecting outcomes such as progression, recurrence, and survival. The purpose of this systematic review was to explore the literature linking distress with survival in solid tumor patients, which may guide future work exploring clinical outcomes as a function of distress.

**Methods:**

A systematic search of PubMed, Embase, and Web of Science was performed using PRISMA (Preferred Reporting Items for Systematic Reviews and Meta‐Analyses) guidelines with predefined eligibility criteria. Thirteen studies met the inclusion criteria and were selected for review.

**Results:**

Findings from this review demonstrated a weak‐to‐moderate relationship between cancer patients' experience of distress and overall survival, with most included studies (11/13) finding at least one predictive analysis to be significant when controlling for confounders. However, significant heterogeneity in the literature, particularly with study sample characteristics and varying methodologies, made direct comparisons across studies challenging.

**Conclusion:**

Findings from this review suggest that psychological distress may have an impact on disease‐related outcomes, including (but not limited to) survival. Future work should consider performing disease‐specific analyses controlling for key prognostic factors to better understand the nuanced relationship between distress and clinical outcomes, which may allow further understanding of the biological underpinnings of this relationship and enable the development of targeted interventions for improving distress.

## Graphical abstract

Key findings from this review demonstrated a weak to moderate relationship between cancer patients’ experience of distress and overall survival, with the majority of included studies finding at least one predictive analysis to be significant when controlling for confounders. This review has significant implications for understanding the existing literature, implications for future study design and methodology, and implications for exploration of shared biologic mechanisms and identification of relevant phenotypes.

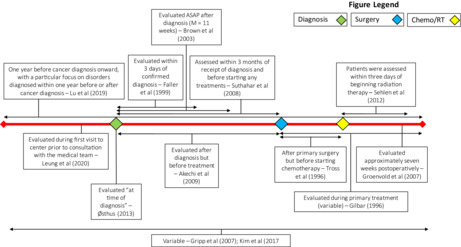

## 1 INTRODUCTION

Research has shown that many cancer patients experience significant psychological distress across the disease trajectory.^1^ In 2014, the American Psychosocial Oncology Society, the Association of Oncology Social Workers, and the Oncology Nursing Society issued a joint statement recommending that the screening and management of distress should be incorporated into routine clinical practice.^2^ They also proposed the global adoption of the National Comprehensive Cancer Network's (NCCN) definition of distress as “a multifactorial unpleasant experience of a psychological, social, spiritual, and/or physical nature that may interfere with the ability to cope effectively with cancer, its physical symptoms, and its treatment”.^3^

While the experience of psychological distress in the general population has been associated with digestive problems, heart disease, sleep problems, mood disturbance, weight gain, and cognitive impairment,^4^ little is known about how this symptom may influence the disease trajectory for cancer patients, affecting outcomes such as progression, recurrence, and survival. Unfortunately, much of the early research examining the relationship between distress and survival has been plagued by severe methodological limitations, including short follow‐up periods, very small sample sizes, and poor control for cancer‐specific prognostic covariates. As a result of these limitations, the literature has conflicting findings regarding the prognostic implications for distress related to survival within oncology.

The purpose of this systematic review is to explore the existing literature on the predictive relationship between psychological distress and survival in solid tumor patients, which may guide future research questions exploring clinical outcomes as a function of distress and may enable the development of targeted interventions to improve this symptom. The guiding research question for this review was as follows: for adult patients with solid tumors, does psychological distress affect patient outcomes as signified by survival?

## 2 METHODS

The methodology for this review, including identification and selection of relevant records, risk of bias assessment, and qualitative synthesis, followed the guidelines set by the Preferred Report Items for Systematic Reviews and Meta‐Analyses (PRISMA) statement checklist.^5^ Studies were selected with the following inclusion criteria: (1) studies assessed the relationship between “distress” (defined at discretion of the researchers) and survival – all types of survival analysis, including overall, progression‐free, disease‐free, and recurrence‐free, were considered; (2) study participants were adults (≥18 years old), with a solid tumor or part of a “mixed” cancer sample consisting of mostly solid tumor patients; (3) studies were in English and used quantitative, qualitative, or mixed methodology. Studies that were not written in English, that included pediatric patients, that included predominantly hematologic cancer patients, or that did not include analysis of the relationship between “distress” and survival were excluded from the review.

**FIGURE 1:**
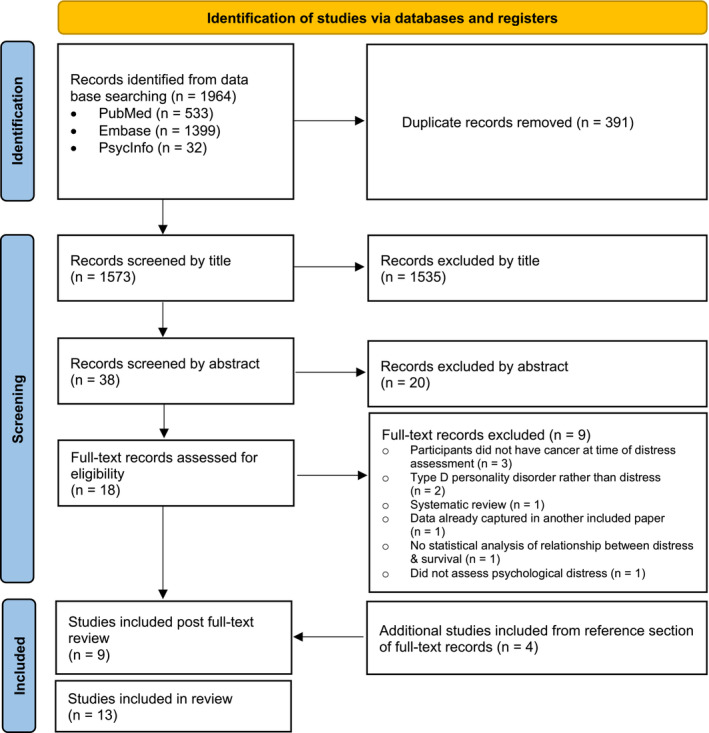
PRISMA flow diagram for systematic review of the literature linking psychological distress with survival in solid tumor patients, which included searches of databases and registers. PRISMA indicates Preferred Reporting Systems for Systematic Reviews and Meta‐Analyses.

A search of PubMed, Embase, and Web of Science was conducted by an expert research librarian (DC) on January 26th, 2021 for studies assessing the impact of distress on survival in adult brain tumor patients, our initial population of interest. This search did not yield any relevant results; therefore, we conducted an expanded search on March 1st, 2021 including all adult solid tumor patients (see full search strategy in Supplement 1). Our search included published, English‐language, peer‐reviewed, original research utilizing qualitative, quantitative, or mixed methodology with no publication year limitations. Two authors (KNR and ALK) performed the initial search, independently screened 1535 titles and 38 abstracts, and reviewed 18 eligible full‐text articles based on eligibility criteria. Of these 18 eligible articles, ninr were determined to satisfy the inclusion criteria. We subsequently screened the reference lists of the nine included articles and identified four additional full‐text articles that met criteria, which resulted in 13 total articles selected for inclusion. Discrepancies were few and predominantly limited to whether the study in question truly assessed the relationship between distress and survival. Such discrepancies were resolved through discussion between KNR, ALK, and third author TSA. Figure 1 details the selection process for included literature.

Each study's risk of bias was independently assessed by two authors (KNR and ALK) based on items from the Cochrane Collaboration Risk of Bias tool^6^ and modified criteria outlined by Tooth et al.^7^ This tool is designed to identify threats to the validity of observational longitudinal studies through the quality assessment of study recruitment, data collection, data analysis, study rationale, study population, and overall generalizability. Discrepancies pertaining to risk of bias assessment for each study were few, limited to clarification of statistical nuances, and were resolved through discussion.

## 3 RESULTS

**FIGURE 2:**
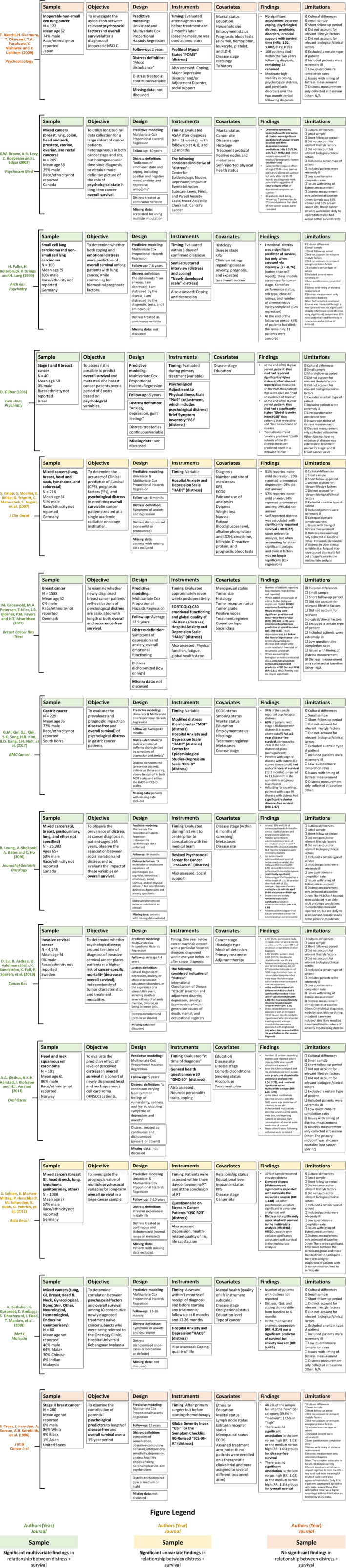
Review of literature assessing the predictive relationship between distress and survival in solid tumor patients. Abstracted data for each study include sample, objective, design, instruments, covariates, key findings, and limitations.

We identified 13 studies assessing the predictive relationship between distress and survival in adult solid tumor patients, encompassing a total of 33,425 patients. Twelve of 13 studies were prospective predictive studies. More than half of the studies had a moderate sample size of approximately 80–200 participants, though sample sizes across the literature ranged from 40 to 25,382 participants, with the largest sample belonging to the single retrospective epidemiological study. Among the pooled participants across studies, 20% had breast cancer, 17% had gastrointestinal cancer, 13% had cervical cancer, 13% had lung cancer, 12% had genitourinary cancer, and the remaining 25% were classified in one of 10 residual categories, including “Other/Not Available” (see Table 1 for additional details on pooled sample characteristics). In addition, five studies were conducted in mixed cancer patients, rather than one specific tumor type. All included studies utilized univariate and/or multivariate Cox proportional hazards regression analyses controlling for pertinent covariates. The majority of studies did not address how missing data were accounted for; however, those that did address missing data did so *via* removal of participants with missing datapoints^8^, ^9^, ^10^, ^11^ or multiple imputation.^12^ Most studies (11/13) found distress to be a significant predictor of survival in at least one predictive analysis when controlling for confounders. Detailed information on all included studies in the review can be found in Figure 2.

**TABLE 1:** Pooled sample characteristics

Pooled sample characteristics		
Geographic location	Number of studies	
North America	3	
Central Europe (Germany)	3	
Scandinavia	3	
East Asia	2	
Middle East (Israel)	1	
Southeast Asia (Malaysia)	1	
Age (year)		
Mean^a^	55	
Range^b^	20–96	
	**Count**	**Percent**
Sex		
Male	13,943	41%
Female	19,736	59%
Tumor type		
Breast	6823	20%
Lung	4457	13%
Colorectal	10	<1%
Head and neck	287	1%
Lymphoma	145	<1%
Gastrointestinal	5536	17%
Gynecological	8	<1%
Genitourinary	4192	13%
Bone and soft tissue	5	<1%
Neurological	1	<1%
Skin	2	<1%
Hematologic	3	<1%
Endocrine	2	<1%
Cervical	4245	13%
Other/Not Available	7709	23%

^a^ Two studies did not provide the mean age of included patients.^11^, ^14^

^b^ Seven studies did not provide the age range of included patients.^10^, ^11^, ^12^, ^14^, ^20^, ^21^, ^25^

### 3.1 Instruments used

While there was significant heterogeneity in distress measurement across the literature, the two most used instruments were the Hospital Anxiety and Depression Scale (HADS) and the Center for Epidemiologic Studies Depression Scale (CES‐D). Four out of 13 studies, including a total of 2113 patients, used the HADS to measure distress.^8^, ^9^, ^13^, ^14^ The HADS is a self‐report tool designed to identify probable cases of depression and anxiety in non‐psychiatric clinical settings; this instrument is comprised of depression and anxiety subscales, each with seven corresponding questions scored on a 4‐point Likert scale. Overall scores range from 0–21, and greater scores indicate higher levels of anxiety and depressive symptoms.^15^ The HADS has demonstrated validity and reliability as a screening tool in oncology, and has indicated particular accuracy when utilizing a cutoff of 9 points to denote possible anxiety disorder and a cutoff of seven points to denote possible depression.^16^, ^17^ Two out of 13 studies, including 434 patients, used the CES‐D to measure distress.^9^, ^12^ The CES‐D is another self‐report tool designed to assess symptoms of depression. Like the HADS, the CES‐D comprises 20 questions, each scored on a 4‐point Likert scale for a total score ranging from 0–60, with greater scores indicative of greater depressive symptomology.^18^ The CES‐D has demonstrated validity and reliability in oncology patients.^19^ Notably, both studies using the CES‐D used this instrument in conjunction with at least one other instrument in their operationalizations of distress.

Each of the remaining studies used a different instrument to measure distress. These instruments vary greatly in focus and methodology, including symptom inventories, like the Brief Symptom Inventory (BSI)^20^ and Symptom Checklist 90‐Revised (SCL‐90‐R)^21^; anxiety questionnaires, like the Lewis, Firich, and Parsell Anxiety Scale^12^; general health questionnaires, like the General Health Questionnaire 30 (GHQ‐30)^22^; events‐based questionnaires, like the Impact of Events Scale (IES)^12^ and Psychological Adjustment to Physical Illness Scale (PAIS)^20^; general mood states questionnaires, like the mood adjective checklist,^12^ Profile of Mood States (POMS),^23^ Revised Psychosocial Screen for Cancer “PSSCAN‐R",^11^ and Questionnaire on Stress in Cancer Patients “QSC‐R23”^10^; as well as interviewing,^24^ a “newly developed [unnamed] scale”,^24^ and searching the medical record for ICD codes related to the psychological disorders.^25^ These instruments are outlined in greater detail in Table 2.

**TABLE 2:** Characteristics of distress instruments used in included articles

Study Instruments											
Instrument	Study	Validated in Oncology	Aspect of Distress Assessed	Cutoff	# of Items	Subscales	Time Window	Examples of Physical Symptoms Assessed	Examples of Feelings Assessed	Examples of Thoughts Assessed	Examples of Behaviors Assessed
Brief Symptom Inventory (BSI)	Gilbar (1996)	Not at time of study; has since been validated^48^	General distress	N/A	53	N/A	Past 7 days	Dizziness, chest pain, nausea, difficulty sleeping	Nervous, annoyed, lonely, fearful, blue, guilt, worthless	Others can control thoughts, suicidal ideation, self‐conscious	Temper outbursts, avoidance
Cantril's Ladder	Brown (2003)	Yes^49^	Sense of control	N/A	2	N/A	Present	None	None	None	None
Center for Epidemiologic Studies‐Depression (CES‐D)	Brown (2003)	Yes^19^	Depressive symptoms	N/A	20	N/A	Past 7 days	Poor appetite, difficulty concentrating, difficulty sleeping	Sad, lonely, fearful	Inferiority, my life is a failure, hopeful for the future	Crying
	Kim (2017)		General distress	No depression symptoms: 0–20 Depression symptoms: ≥ 21							
General Health Questionnaire 30 (GHQ‐30)	Osthus (2013)	Yes^50^	General distress	Low distress: < mean GHQ sum score (30) High distress: > mean GHQ sum score (30)	30	N/A	Present	Difficulty concentrating, difficulty sleeping, restless	Unhappy, depressed, nervous	Worthless, hopeless, life not worth living	Enjoy normal activities, playing useful part, making decisions, not busy, not leaving the house, not getting along with others
Hospital Anxiety and Depression Scale (HADS	Gripp (2007)	Yes^50^	General distress	None/mild depression: ≤10 Pronounced depression: >10 None/mild anxiety: ≤10 Pronounced anxiety: >10	14, 7 for each subscale	2: Anxiety; Depression	Past 7 days	None	Frightened, cheerful, relaxed, tense, panic	Worrying thoughts, hopeful for future	Enjoy normal activities, loss of interest in appearance
	Groenvold (2007)		General distress	Low depression: 0–7 or 0–10 High depression: 8–21 or 11–21 Low anxiety: 0–7 or 0–10 High anxiety: 8–21 or 11–21							
	Kim (2017)		General distress	No distress: 0–7 Distress: 8–21							
	Suthahar (2008)		General distress	Depression non‐case: ≤7 Depression borderline case: 8–10 Depression definite case: ≥11							
Impact of Events ‐ Intrusion Subscale Only (IES‐R)	Brown (2003)	Yes^51^	Adverse life events	N/A	7 (intrusion subscale only)	N/A	Past 7 days	Difficulty sleeping	Strong feelings	Recurrent thoughts and dreams about stressful event	Avoidance
International Classification of Disease Codes (ICD‐10)	Lu (2019)	N/A	Clinical diagnoses of stress reaction & adjustment disorder, depression, and/or anxiety disorder	N/A	N/A	N/A	One year preceding cancer diagnosis onwards	None	None	None	None
Lewis, Firsich, and Parsell Anxiety Scale	Brown (2003)	Yes^52^	Anxiety symptoms	N/A	9	3: Nausea/vomiting; body regard; anxiety	Present	Difficulty sleeping	Nervous, irritable, upset, tense, jittery, anxious	Having nightmares	Cannot sit still
Modified Distress Thermometer (MDT)	Kim (2017)	Yes^53^	General distress	Scores >4 points in both the severity and impairment scales considered indicative of need to refer to a psychiatrist	1 item per subscale	3: insomnia; anxiety; depression	Unknown	Fatigue, nausea, difficulty sleeping	Nervousness, worry, fears	Loss of faith, loss of sense of purpose	Loss of interest in usual activities
Mood Adjective Check List (only some items used)	Brown (2003)	Yes^54^	Pleasant and unpleasant mood	N/A	130 (only 10 items used)	2: Positive mood; Negative mood	Unknown	Unknown	Unknown	Unknown	Unknown
Newly Developed Scale	Faller (1999)	Unknown	General distress	N/A	Unknown	Unknown	Unknown	Unknown	Unknown	Unknown	Unknown
Profile of Mood States (POMS)	Akechi (2009)	Yes^55^	General distress	N/A	65	2: Negative (tension, depression, fatigue, confusion, anger); Positive (vigor and esteem‐related affect)	Past 7 days	Difficulty concentrating	Angry, unhappy, sad	Worthless, hopeless	None
Psychological Adjustment to Physical Illness Scale (PAIS)	Gilbar (1996)	Not at time of study; has since been validated^56^	Adjustment	N/A	46	7: Healthcare orientation; Vocational environment; Domestic environment; Sexual relationships; Family relationships; Social environment; Psychological distress	During treatment (variable)	Unknown	Unknown	Unknown	Unknown
Questionnaire on Stress in Cancer Patients (QSC‐R23)	Sehlen (2012)	Yes^57^	General distress	Mean scores >1.5 considered cases	23	5: Psychosomatic complaints; Fears; Information deficits; Everyday life restrictions; Social strains	Present	Tired, weak, difficulty sleeping	Nervous, tense	Fear of progression, fear of pain, fear of going to hospital, feeling physically imperfect	Going out less, having sex less frequently
Revised Psychosocial Screen for Cancer (PSSCAN‐R)	Leung (2020)	Yes^58^	General distress	Minimal distress: <8 Subclinical depression and anxiety: 8–10 Clinical depression and anxiety: ≥11	21	N/A	Some 30 days, some 7 days, some present	General physical health, heart racing	Nervous, on edge, restless, tense, depressed	Repetitive scary thoughts, suicidal ideation	Loss of interest in activities, unable to work or carry out activities
Semi‐structured interview	Faller (1999)	N/A	Coping	N/A	N/A	N/A	Unknown	None	None	None	None
Symptom Checklist 90‐Revised (SCL‐90‐R)	Tross (1996)	Yes^59^	General distress	Low distress: raw GSI scores <0.38 Medium distress: raw GSI score 0.38–0.90 High distress: raw GSI score ≥0.91	90	9: Somatization; Obsessive–compulsive; Interpersonal sensitivity; Depression; Anxiety; Hostility; Phobic anxiety; Paranoid ideation; Psychoticism	Past 7 days	Headache, dizziness, chest pain, nausea, difficulty sleeping	Nervous, annoyed, lonely, fearful, blue, guilt, worthless	Others can control thoughts, suicidal ideation, self‐conscious	Temper outbursts, avoidance, overeating

### 3.2 Timing of distress measurement

**FIGURE 3:**
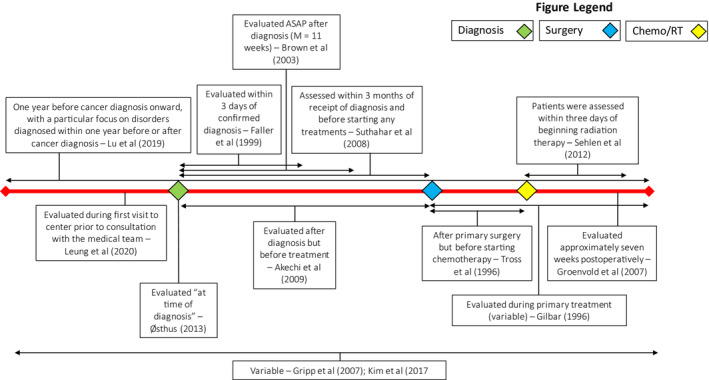
Timing of distress measurement across the disease trajectory for included articles. Figure 3 illustrates the windows that each study included in this review measured distress within. Three major timepoints in the clinical course – time of diagnosis, time of surgery, and time of chemotherapy and/or radiation treatment are marked on the timeline for comparison.

In addition to significant heterogeneity in the instruments used to measure distress, there was also significant variation in the time at which distress was measured; Figure 3 further illustrates these timepoints and windows. Five studies aimed to measure distress relative to initial diagnosis^12^, ^14^, ^22^, ^23^, ^24^; however, these studies varied considerably in how closely the assessment of distress followed the diagnosis. Four studies^10^, ^13^, ^20^, ^21^ assessed distress at timepoints relative to primary surgery or the initiation of radiation and/or chemotherapy. One study assessed distress up to 1 year before diagnosis onwards,^25^ while another assessed distress during first visit to the medical center prior to consultation with the medical team.^11^ The remaining two studies assessed distress at variable timepoints not further specified.^8^, ^9^

### 3.3 Main findings by cancer type

A few studies looked at similar cancer types in their analyses, which allowed direct comparison of findings.

#### 3.3.1 Breast cancer

Three studies examined the relationship between distress and survival in breast cancer patients. Two of these studies^13^, ^20^ found distress to be a significant predictor of survival in univariate analysis only. The study by Gilbar^20^ had a much smaller sample size (40 patients), evaluated distress at variable timepoints throughout treatment using the BSI, and only controlled for disease stage and education level. In contrast, Groenvold et al.^13^ had a larger sample size of 1588 patients, as well as a consistent timing of distress assessment using the HADS at the 7‐week postoperative timepoint. Furthermore, this study controlled for several breast cancer‐specific clinical covariates, including menopausal status and positive nodes. The third study in breast cancer patients found no significant results in any analysis.^21^ This study had a sample size of 280 patients, and, similar to Groenvold et al.,^13^ had relatively consistent timing of distress assessment, while also including several relevant clinical covariates. Tross et al.^21^ was unusual, however, in defining distress as inclusive of several distinct constructs of psychopathology, including obsessive–compulsive behavior, phobic anxiety, psychoticism, and paranoid ideation; this is in contrast to Gilbar^20^ and Groenvold et al.,^13^ where distress was considered more strictly related to symptoms of depression and anxiety.

#### 3.3.2 Lung cancer

Of the two studies examining the predictive relationship between distress and survival in lung cancer patients, one study found no significant results,^23^ while the other found that distress predicted survival when measured *via* interview, but not *via* self‐report.^24^ The sample sizes in these two studies were comparable (122 and 103 patients, respectively), and in both studies approximately 89% of patients had died by the end of the follow‐up period. However, the follow‐up period in Akechi et al.^23^ was much shorter at 2 years than at 7–8 years in Faller et al.^24^ Furthermore, this study was conducted in Japan and utilized the POMS to measure distress between the time of diagnosis and treatment. The study conducted by Faller et al.^24^ was conducted in Germany and measured distress using a “newly developed” (otherwise unidentified) self‐report scale and *via* interview, evaluating patients strictly within 3 days of receipt of diagnosis. Although these two studies appear to have defined distress in a relatively similar way (as “mood disturbance” and by statements such as “I feel anxious/depressed/distressed”), the potential cultural differences in distress reporting across Japan and Germany, differences in length of follow‐up, and the use of a “newly developed” scale to measure distress may have contributed to disparate findings.

#### 3.3.3 Other solid tumors

The remaining three tumor‐specific studies were conducted in patients with invasive cervical cancer,^24^ head and neck cancer,^22^ and gastric cancer.^9^ All three of these studies found that distress was predictive of survival in multivariate analysis, although each used a different instrument to measure distress. The studies were also highly variable in terms of sample size (ranging from 101 to 4245), covariates controlled for, and in the timing of distress assessment, with evaluations taking place 1 year prior to cancer diagnosis onwards, at time of diagnosis, and variably throughout the cancer trajectory. Additionally, each of these three studies had similar definitions of distress that were largely characterized by depression and anxiety. However, while Lu et al.^26^ focused on identifying a clinical diagnosis of adjustment, major depressive, or anxiety disorders (in addition to documenting stressful life events), Østhus et al.^22^ and Kim et al.^9^ focused on symptoms of depression and anxiety, as measured by the GHQ‐30 and the HADS, respectively.

#### 3.3.4 Mixed cancers

Five studies, including a total of 26,717 patients, examined the predictive relationship between distress and survival in a sample of “mixed” cancer patients, with no additional analyses based on cancer subtype. The majority of these patients (94%) were enrolled in the study conducted by Leung et al.,^27^ which only included geriatric oncology patients aged 65 and older. Among the pooled mixed cancer samples, 20% of patients had GI cancer; 18% had breast cancer; 16% had genitourinary cancer; 16% had lung cancer; and 29% were classified as having a “other” cancer. Two of the five mixed cancer studies^12^, ^14^ found that only depressive symptoms (as measured by the CES‐D and the HADS, respectively) were predictive of overall survival; one study found that distress was only a significant predictor of OS in univariate analysis^8^; another study found significant multivariate results^27^; and the last study did not report any significant results for distress predicting survival.^10^

### 3.4 Risk of bias assessment

The risk of bias results for all included studies are provided in Table 3. Based on our risk of bias assessment, all included studies were at moderate to high risk of bias, with the most concerning study aspects including selection bias, lacking justification for numbers of participants, no account for lost to follow‐up in analysis, lacking consideration for missing data, and unclear or missing comparisons between consenters and non‐consenters. On average, each study answered ‘yes’ (low risk) to 21 of the 32 included risk of bias questions. The authors chose to exclude the assessment question “was the validity (against a gold standard) of measurement methods mentioned?” since there is not yet a consensus “gold standard” distress instrument within psycho‐oncology.^28^

**TABLE 3:** Cochrane risk of bias assessment with modified criteria by Tooth et al.^7^ for included literature

Question	Akechi (2009)	Brown (2003)	Faller (1999)	Gilbar (1996)	Gripp (2007)	Groenvold (2007)	Kim (2017)	Leung (2020)	Østhus (2013)	Tross (1996)	Lu (2019)	Suthahar (2008)	Sehlen (2012)
Are the objectives or hypotheses of the study stated?	+	+	+	+	+	+	+	+	+	+	+	+	+
Is the target population defined?	+	+	+	+	+	+	+	+	+	+	+	+	+
Is the sampling frame defined?	+	+	+	+	+	+	+	+	+	+	+	+	+
Is the study population defined?	+	+	+	+	+	+	+	+	+	+	+	+	+
Are the study setting (venues) and/or geographic location stated?	+	+	+	+	+	+	+	+	−	−	+	+	+
Are the dates between which the study was conducted stated? (rather than implicit)	+	−	+	+	+	+	+	+	+	+	+	+	+
Are eligibility criteria stated?	+	+	+	−	−	−	+	−	+	−	+	+	+
Are issues of “selection in” to the study mentioned?	+	−	−	−	−	−	−	−	−	−	−	−	+
Is the number of participants justified?	−	−	−	−	−	−	−	−	−	−	−	−	−
Are numbers meeting and not meeting the eligibility criteria stated?	+	−	−	−	+	+	−	−	−	−	+	+	+
For those not eligible, are the reasons why stated?	+	−	−	−	+	−	−	−	−	−	+	+	+
Are the numbers of people who did/did not consent to participate stated?	+	+	+	−	+	+	−	−	+	+	−	+	+
Are the reasons that people refused to consent stated?	−	−	−	−	−	−	−	−	−	−	−	−	−
Were consenters compared with nonconsenters?	+	+	+	−	−	−	−	−	−	−	−	−	+
Was the number of participants at the beginning of the study stated?	+	+	+	+	+	+	+	+	+	+	+	+	+
Were methods of data collection stated?	+	+	+	+	+	+	+	+	+	+	+	+	+
Was the reliability (repeatability) of measurement methods mentioned?	+	+	+	+	−	−	+	−	+	+	−	−	+
Was the validity (against a “gold standard”) of measurement methods mentioned?	N/A	N/A	N/A	N/A	N/A	N/A	N/A	N/A	N/A	N/A	N/A	N/A	N/A
Were any confounders mentioned?	+	+	+	+	+	+	+	+	+	+	+	+	+
Was the number of participants at each stage/wave specified? (i.e. baseline and EOS)	+	+	+	+	+	+	−	−	+	+	+	+	+
Were reasons for loss to follownup quantified?	−	−	−	−	−	−	−	−	−	−	−	−	−
Was the missingness of data items at each wave mentioned?	−	+	−	−	+	−	+	+	−	−	−	−	+
Was the type of analyses conducted stated?	+	+	+	+	+	+	+	+	+	+	+	+	+
Were “longitudinal” analysis methods stated?	+	+	+	+	+	+	+	+	+	+	+	+	+
Were absolute effect sizes reported?	−	−	+	−	−	−	+	+	−	+	−	−	+
Were relative effect sizes reported?	+	+	+	−	+	+	+	+	+	+	+	+	+
Was loss to follow‐up taken into account in the analysis?	−	+	−	−	−	−	+	−	−	−	−	−	−
Were confounders accounted for in analyses?	+	+	+	−	+	+	+	+	+	+	+	+	+
Were missing data accounted for in the analyses?	−	+	−	−	+	−	+	+	−	−	−	−	+
Was the impact of biases assessed qualitatively?	+	+	+	−	+	+	+	+	+	+	+	+	+
Was the impact of biases estimated quantitatively?	−	−	+	−	−	−	−	−	−	−	−	−	−
Did authors relate results back to a target population?	+	+	+	+	−	+	+	+	+	+	+	+	+
Was there any other discussion of generalizability?	+	+	+	+	+	+	+	+	+	+	+	+	+

*Note*: Table 3 summarizes results from the Cochrane risk of bias with modified criteria by Tooth et al.^7^ assessment. Fields marked with a “+” indicate that the answer to the corresponding question for that study was “yes” (low risk). Fields marked with a “−” indicate that the answer to the corresponding question for that study was “no” (high risk). The question about validity verification against a gold standard is marked as “N/A” as there is no consensus on a gold standard instrument for measurement of psychological distress.

## 4 DISCUSSION

This systematic review included 13 studies assessing the impact of psychological distress on survival in solid tumor patients. There was a great deal of variation in how distress was defined and measured across studies, with anxiety and depressive symptoms being the most common indicator of distress. All but two of the included studies reported a predictive relationship between distress and survival in at least one analysis. Both studies reporting null findings had relatively low distress reporting in their samples, which may be indicative of floor effects, hindering the demarcation of “distressed” versus “non‐distressed” patients and making statistically significant relationships difficult to detect. The sample in Akechi et al.^23^ had a median Total Mood Disturbance (TMD) score of 27 on the POMS, with possible scores ranging between −24 and 177 and the TMD in the general population estimated to be around 15–20 with a standard deviation of 33.^29^ This relatively low reporting of distress in a Japanese sample is in stark contradiction to other reports that the percentage of lung cancer patients experiencing clinically significant distress is upwards of 50% using the Distress Thermometer (DT) and higher than any other cancer type, including brain, pancreatic, breast, and prostate cancers, when using the BSI.^30^, ^31^ Furthermore, only 12.5% of the sample in Tross et al.^21^ was classified into the ‘high distress’ category when utilizing the Global Severity Index (GSI) of the Symptom Checklist 90‐Revised, again despite the fact that the proportion of breast cancer patients experiencing clinically significant distress has been estimated in other work to be around 25% at time of diagnosis and 17% at time of primary surgery when utilizing the BSI.^32^

Both studies reporting null findings had at least one counterpart study included in our review that was conducted in the same cancer type but did find a significant predictive relationship between distress and survival. These discrepancies may underscore the need for greater standardization in the way that distress is measured within oncology. Interestingly, comparing two of the three studies conducted in breast cancer patients, Tross et al.^21^ used the GSI of the SCL‐90R and found null results, while Gilbar^20^ found that a higher GSI on the BSI was significantly associated with shorter survival. While the burden of utilizing the longer SCL‐90R as opposed to the BSI may have contributed to the disparate findings of these two studies to a degree, it is important to note that Gilbar et al.^20^ only accounted for one clinical covariate in their analysis. Therefore, there may be underlying clinical or demographic variables mediating the emergent relationship between distress and survival identified in this study.

Surprisingly, none of the 13 studies included in this review utilized the Distress Thermometer (DT) to measure clinically significant distress in cancer patients, which is a self‐report tool recommended for use in oncology patients by the NCCN.^3^ The DT was designed to rapidly assess distress, minimizing burden on patients and thereby facilitating repeat assessments. The DT has been estimated to take 2 minutes and 20 seconds to accurately administer.^33^ Patients rate their experience of distress over the past 7 days on a scale of 1 to 10, identifying the source of distress through an accompanying problem list. The DT has been widely accepted as a valid and reliable tool to assess distress and has been translated and validated in over 18 non‐English languages.^34^ Given its widespread use in clinical settings, the DT may be an ideal measurement tool to standardize distress measurement across studies and allow comparison of findings within similar cancer populations.

When comparing results across cancer types, discrepancies in findings may suggest that the manifestation and impact of distress on survival may vary in different oncology populations. For example, cancers with better treatment options, in which the patient is more likely to live with the after‐effects of cancer and treatment, may invoke high levels of distress without that distress impairing survival. One study in post‐treatment breast cancer patients, for example, found that fear of recurrence was a significant concern for patients several years after primary diagnosis, even in patients with a relatively high quality of life and satisfaction with treatment.^35^ On the other hand, patients with advanced cancers are more likely to be very ill and thus may be more challenging to assess. Distress in terminal stages of life may also vary uniquely as a function of factors relevant to end‐of‐life care, such as acceptance of prognosis and perceived burden on caregivers.^36^, ^37^ In addition, very ill patients are less likely to be included in studies—some of the studies included in this review, for example, excluded patients with metastases, severe illness, and cognitive impairment, which may bias the true prevalence and impact of distress on survival.

Another variable that was inconsistent across studies was the timing of distress measurement. A review study conducted by Ziegler et al.^38^ suggested that the sensitivity of distress instruments may vary depending on the stage of illness trajectory they are used in. In this study, the HADS was identified as superior for recognizing distress during pre‐treatment and post‐treatment, the HADS and Mental Health Inventory‐5 (MHI‐5) together were identified as superior for recognizing distress during treatment, and the Brief Edinburgh Depression Scale (BEDS) was identified as superior for recognizing distress during recurrence and palliative care. This variation in instrument performance may reflect the fact that distress levels and symptomology fluctuate as a function of the cancer illness trajectory, thereby making comparison of studies with different distress measurement timing windows difficult.

An intriguing aspect of the articles included in this review is that the studies were conducted in several culturally and geographically distinct locations. A few articles discussed study results in the context of the country they were conducted in: for example, Groenvold et al.^13^ highlighted that the relationship between distress and survival may be stronger since the study was conducted in a small homogenous country (Denmark) where perceptions of distress are likely to be more consistent across patients than in large, diverse countries. Furthermore, Akechi et al.^23^ highlighted that in Japan, the practice of disclosing a cancer diagnosis to patients is not universally practiced, and therefore cultural differences in patient–physician communication and the social meaning of a cancer diagnosis may have influenced the reporting of distress. The impact of culture and perceptions surrounding psychological distress within a particular country or region has rarely been studied within oncology and highlights an important gap in the literature that warrants exploration.

For those studies that found a significant predictive relationship between distress and survival, this finding might be explained in two ways: (1) through impact of distress on pro‐health behaviors that support improved general health status and recovery from cancer and associated treatments; (2) through direct biologic pathways implicated in both distress and tumor growth.

In the general population, increased levels of distress have been associated with poor health behaviors, including decreased fruit and vegetable intake, decreased physical activity, increased sedentary lifestyle, and increased tobacco use, with many of these findings replicated in cancer patients and survivors.^39^ In a sample of head and neck cancer patients, increased psychological distress was a significant determinant of which patients continued to smoke after cancer diagnosis.^40^ Additionally, in a sample of prostate cancer survivors, individuals not compliant with contemporary exercise‐oncology guidelines had significantly higher global distress and anxiety as measured by the BSI.^41^ Some research has estimated that physical inactivity, poor diet, and tobacco use are implicated in approximately 30% of all cancer‐related deaths, underscoring the importance of these pro‐health behaviors that distressed cancer patients may disproportionately not be engaging in.^42^

Furthermore, the experience of psychological distress may have a direct impact on the mechanisms relating to tumorigenesis and disease progression. A recent review of preclinical and clinical models of cancer and distress found that distress may affect several aspects of the disease process, including development of disease via catecholamine‐ and glucocorticoid‐mediated inhibition of DNA repair and β‐adrenergic receptor feedforward tumorigenesis; disease progression though catecholamine promotion of tumor proliferation, survival, invasion, and migration; and treatment resistance through impaired immune cell functioning.^43^, ^44^, ^45^ Additional studies have even suggested a possible cyclic effect of psychological distress on cancer outcomes. Aldea et al.^46^ suggested that cancer promotes inflammation and cytokine production, which have been associated with depression, and conversely, depression promotes hypothalamic–pituitary–adrenal axis (HPA) dysfunction, which further promotes immune function strain and increased cytokine production.^46^ Nonetheless, further research is needed to clarify the mechanisms by which distress may interplay with tumorigenesis, progression, and treatment, especially as they relate to cancer‐specific clinical models of disease.

## 5 LIMITATIONS

There are several limitations to this review. It is possible that limiting our search to English‐language publications and using “cancer” and “solid tumors” as search terms (rather than specific disease names) may have resulted in the unintended exclusion of relevant literature. Additionally, given that the initial population of interest was brain tumors, many of the search criteria focus on this tumor type. While this may have potentially weighted search results away from other solid tumors when the scope of the review was expanded, we found no papers exclusively looking at distress within the PBT population. The significant heterogeneity in the included literature in terms of sample characteristics as well as conceptualization and measurement of distress makes drawing concrete conclusions about the impact of distress on survival difficult, given that these factors are likely an integral part of the larger picture encompassing both distress and clinical outcomes. In addition, several of the included studies were conducted in distinct cultures and geographic regions. Our inability to account for cultural differences in the experience and reporting of distress is yet another limitation of this review. Last, a few studies included in our review found significant results, but only in univariate analysis. Given that cancer survival is largely influenced by clinical and demographic variables, the results of these studies should be interpreted with caution. Nevertheless, our findings are compelling and underscore the need for additional research in this field.

## 6 CONCLUSION

This review highlights evidence of a weak‐to‐moderate relationship between cancer patients' experience of distress and overall survival, with the majority of included studies finding at least one predictive analysis to be significant. However, substantial discrepancies in how distress is defined, measured, and addressed in various cancer populations make drawing further definitive conclusions difficult. Nonetheless, research has shown that the experience of psychological distress among cancer patients is pervasive and often undermanaged.^47^ Based on these results, future studies should consider performing disease‐specific analyses, inclusive of biologic covariates, in order to better understand the nuanced relationship between distress and cancer patients' clinical outcomes. In doing so, we may better understand the clinical implications of untreated distress and enable the development of targeted interventions to improve outcomes for those who are most likely to experience it.

## AUTHOR CONTRIBUTIONS

Kayla Roche, Dr. Armstrong, and Dr. King conceived the study design for the review. Diane Cooper conducted the initial literature search. Ms. Roche and Dr. King participated in identifying and reviewing the included literature, and Ms. Roche drafted the initial manuscript. Dr. Armstrong and Dr. King both provided helpful feedback during the manuscript revision process. All authors have reviewed and approved the final version of this manuscript and these data have not been published previously, either in whole or in part.

## CONFLICT OF INTEREST

The authors have no conflicts of interest to disclose.

## DISSEMINATION

This research has not been presented or published elsewhere.

## ETHICS STATEMENT

Ethical approval was not sought for this article; in being a systematic review, only pre‐existing data were analyzed in this study.

## Data Availability

Data sharing is not applicable to this article as no new data were created or analyzed in this study.
